# Determination of the Optimal Dose Reduction Level via Iterative Reconstruction Using 640-Slice Volume Chest CT in a Pig Model

**DOI:** 10.1371/journal.pone.0117213

**Published:** 2015-03-12

**Authors:** Xingli Liu, Jingshi Wang, Qin Liu, Pengfei Zhao, Yang Hou, Yue Ma, Qiyong Guo

**Affiliations:** 1 Department of Radiology, Shengjing Hospital of China Medical University, 36 Sanhao Street Shenyang, Liaoning Province, PR China 110004; 2 Department of cardiology, Shengjing Hospital of China Medical University, 36 Sanhao Street Shenyang, Liaoning Province, PR China 110004; 3 Department of Radiology, Dalian Municipal Women and Children’s Medical Central, 1 Guihua Street, Dalian, Liaoning Province, PR China 116033; University of Iowa, UNITED STATES

## Abstract

**Aim:**

To determine the optimal dose reduction level of iterative reconstruction technique for paediatric chest CT in pig models.

**Materials and Methods:**

27 infant pigs underwent 640-slice volume chest CT with 80kVp and different mAs. Automatic exposure control technique was used, and the index of noise was set to SD10 (Group A, routine dose), SD12.5, SD15, SD17.5, SD20 (Groups from B to E) to reduce dose respectively. Group A was reconstructed with filtered back projection (FBP), and Groups from B to E were reconstructed using iterative reconstruction (IR). Objective and subjective image quality (IQ) among groups were compared to determine an optimal radiation reduction level.

**Results:**

The noise and signal-to-noise ratio (SNR) in Group D had no significant statistical difference from that in Group A (P = 1.0). The scores of subjective IQ in Group A were not significantly different from those in Group D (P>0.05). There were no obvious statistical differences in the objective and subjective index values among the subgroups (small, medium and large subgroups) of Group D. The effective dose (ED) of Group D was 58.9% lower than that of Group A (0.20±0.05mSv vs 0.48±0.10mSv, p <0.001).

**Conclusions:**

In infant pig chest CT, using iterative reconstruction can provide diagnostic image quality; furthermore, it can reduce the dosage by 58.9%.

## Introduction

Due to revolutionary improvements in the temporal and spatial resolution, multi-slice computed tomography (MSCT) has gained increasingly widespread applications for children.[1, 2] It was estimated that in 2007 CT examinations for children accounted for 10% of the total CT scans performed in the USA; moreover, this number could reach 16–20% in developing continents like Asian and African.[1, 2] However, due to the immaturity of their organs and their longer future lifetime, children could have a much higher risk in radiation-induced cancer.[3] Therefore, extreme cautions should be exercised in exposing children to CT radiation, and appropriate criteria for complicated thoracic lesions (e.g, tumours, tracheal dysplasia, severe pulmonary infection, abscesses, and chest trauma) should be well established. In addition, paediatric chest MSCT must adhere to the as low as reasonably achievable (ALARA) principle.

To date, low-dose techniques applied to paediatric chest CT primarily include individualization of body weight (or body size) adaptive scanning parameters, reduction of tube current and voltage, automatic exposure control (AEC), selective organ shielding, and decreasing the field of view (FOV).[4–10] These methods can reduce the computed tomography dose index (CTDI) to 3.5–9.5 mGy for chest CT in infants and children.[11] However, the traditional filtered back projection (FBP) reconstruction techniques require a high number of photons for imaging; thus, it can further restrict the dosage reduction.

Iterative reconstruction (IR) algorithms are recent developments in CT reconstruction, and their application to CT examinations of the adult chest has proven that IR can significantly reduce noise induced by low-dose scanning; thus, it can reduce the radiation dose by 64.2–80%.[12–14] However, there are few available data regarding the application of a paediatric chest CT. Lee et al. [15] showed that the application of adaptive statistical iterative reconstruction (ASIR) to a paediatric chest CT could reduce the radiation dose by 55% without impacting the image quality. Miéville et al. [16] conducted a study on low-dose CT in children with pulmonary cystic fibrosis; they found that that model-based iterative reconstruction (MBIR) has the potential to significantly reduce radiation dosage, while maintaining the visibility of small pulmonary structures.[16] According to the ALARA principle, the optimal dose level and scanning scheme for paediatric chest CT using IR algorithms need to be determined. Since the young pigs have a similar cardiothoracic structure as that of human infants, pigs were selected on the basis of weight in order to imitate the thoracic conditions of human infants aged 0–3 years. In this paper, we aimed to study the potential of reducing the radiation dose in infant pig chest CT using an IR algorithm, adaptive iterative dose reduction 3D (AIDR-3D) reconstruction and to investigate the optimal dose reduction levels in order to maintain the diagnostic image quality.

## Materials and Methods

### 1. Objects and Animal Preparation

Thirty infant pigs aged 0–2 months with a mean weight of 7.52 ± 2.75 kg (range: 3.5–12 kg) were selected. According to the Reference Standard for Growth and Development of Chinese Children below 7 Years Old (2009 Edition), the pigs were divided into three subgroups with 10 selected pigs in each group, based on body weight (small subgroup: body weight ≤ 5kg; medium subgroup: 5 kg < body weight < 10kg; large subgroup: 10kg ≤ body weight ≤15kg). Each pig first underwent a chest CT at a routine dose and then at four different low-dose levels. All animal experimental procedures were approved and performed under the supervision of the ethics committee of the ShengJing Hospital of China Medical University (Shenyang 110004, China, no. 2012PS35K).

After fasting without food and water for 12 hours, the pigs received an intraperitoneal injection of 0.4 ml/kg xylazine hydrochloride (1.5 ml (30 mg), Jilin Huamu Animal Health Product Co., Ltd.) to induce anaesthesia. Additional anaesthesia of an intraperitoneal injection of 0.5 ml (for every additional injection) was prepared in case that the subject regained consciousness during the procedure.

### 2. CT Acquisition and Reconstruction

A 640-slice volume CT (Aquillion ONE; Toshiba Medical Systems Corporation, Japan) was conducted for each chest CT. The pigs were fixed in the supine position after anaesthesia had been established. Each pig underwent five CT procedures composing of a routine-dose and four low-dose scans. The automatic exposure control (^SURE^Exposure 3D) and volume scanning mode were used. First, the routine-dose scan (80 kVp, SD10 with FBP reconstruction) was performed for Group A. Then, the tube voltage was fixed at 80 kVp and the tube current was reduced by gradually increasing the noise index (SD12.5 (Group B), SD15 (Group C), SD17.5 (Group D), and SD20 (Group E)); IR reconstruction was conducted in Group B-E (AIDR-3D at the strong dose reduction level). Other scan parameters of Group A to E were kept the same as follows: ^SURE^Exposure 3D; tube current of 15–115 mAs; rotation time of 0.5 s; collimator of 0.5 mm; pitch of 0.844; slice thickness of 2 mm; slice increment of 2 mm, and the field of view of 120–160 mm. The images of the mediastinal and lung windows were reconstructed using FC17 and FC55 convolution kernels respectively. The window width/window position was set at 1500 HU/-500 HU for the lung window and 400 HU/40 HU for the mediastinal window.

### 3. Image Analysis

#### 3.1. Evaluation of Objective Image Quality

All images were transferred to a Aquillion ONE workstation (Toshiba Medical System Corporation, Japan). The objective indices included the CT value of the descending aorta (CTAO), its noise (Noise_AO_), and its signal-to-noise ratio (SNR). A region of interest (ROI) of 100 mm^2^ was set in the descending aorta at the level of the tracheal bifurcation in the mediastinal window. The mean CT value (CTAO mean) of the descending aorta in the ROI was measured, and its standard deviation (SD) was defined as the image noise (NoiseAO_mean). The SNR was calculated using the formula: SNR = CT_AO mean_/Noise _AO mean_.

All objective indices were calculated by an experienced radiologist who was blinded to any information. The mean value of three continuous slices was calculated for all of these objective indices.

#### 3.2. Analysis of Subjective Image Quality

Images of the five groups were evaluated by two experienced radiologists in a blind manner. The evaluation indices and scoring standards were selected by referring to CT image quality guidelines recommended by European standards.[17–19] The evaluation indices were as follows: (1) characteristics of the central airway (trachea, lobar bronchi, and segmental bronchi in the lung window); (2) manifestations of lung tissue in the lung window; (3) mediastinal structures (characteristics of the mediastinum, pleura, and other soft tissues in the mediastinal window); (4) artefacts in both the lung window and the mediastinal window (respiratory motion artefact, stripe artefact, and/or a plastic appearance); (5) general image quality (comprehensive evaluation of the image quality of the lung and the mediastinal windows). All these indices were scored using a 5-point scale, where 1 was classified as very poor; 2 was poor; 3 was fair; 4 was good; 5 was excellent. The images with scores ≥ 4 were considered adequate to meet clinical requirements. If necessary, to obtain a consensus score, the opinion of a third radiologist was referred to adjudicate differences.

### 4. Evaluation of Radiation Dose

CT dose index volume (CTDI_vol_) and dose length product (DLP) were recorded after scanning; the effective dose (ED) was calculated according to the formula of ED = DLP×κ, where κ is the conversion coefficient for paediatric chest CT. The κ value was set to 0.039 when the pig’s weight was ≤ 5 kg, and it was set to 0.026 when the pig’s weight was 5–15 kg.[20]

### 5. Statistical Analysis

Statistical analysis was performed using commercially available software (SPSS 17.0; SPSS, Chicago, IL, USA). Continuous variables were expressed as Mean ± SD. The kappa test was used to test inter-reader agreement in subjective evaluation of image quality, and the consensus score was used for the final result. Objective indices (CT_AO mean_, Noise _AO mean_ and SNR) and radiation dose (CTDI_vol_, DLP and ED) in each group were analysed by randomized block analysis of variance. Subjective indices were analysed by the Friedman test. Multiple comparisons were performed by the Bonferroni test. Objective and subjective indices of subgroup were analysed by the ANOVA and Kruskal-Wallis test. *P* < 0.05 was considered to be statistically significant.

## Results

Three pigs expired while under anaesthesia, and the remaining 27 were successfully anesthetized to undergo CT scanning. The general information about the pigs is presented in Table 1.

**Table 1:** General data of the swine at three Body Weight level.

Item	Small (n = 9)	Medium (n = 8)	Large (n = 10)
Body Weight (kg)	4.7±0.5(3.5–5.0)	8.2±1.3(6.0–9.5)	12.4±2.3(10.0–15.0)
Antero-posterior Diameter of Thorax (mm)	97.8±9.6(83.4–113.8)	109.3±13.4(85.2–120.0)	119.6±16.9(83.0–141.9)
Transverse Diameter of Thorax (mm)	120.9±9.3(106.7–134.7)	130.7±6.0(123.8–138.7)	137.8±15.5(112.2–155.7)
Scan Length (cm)	13.2±1.4(10.0–14.0)	13.3±0.9(12.0–14.0)	14.5±0.9(14.0–16.0)

Note: Small subgroup: Body Weight ≤ 5kg; Medium subgroup: 5 kg < Body Weight < 10kg; Large subgroup: 10kg ≤ BodyWeight ≤15kg

### 1. Objective Image Quality Comparison

The comparison of objective image quality among the groups was presented in Table 2. There were no significant statistical differences in the CTAO _mean_ value among the groups (*P* = 0.76). There were significant statistical differences in the NoiseAO _mean_ value among the groups (*P* < 0.001), and the noise of the aorta in Groups B and C were significantly lower than that of Group A. There were no statistical differences in Noise_AO mean_ between Groups A V.S D and Group A V.S E. Compared with Group A, the SNR in Groups B and C was increased (*P* < 0.05). The SNR of Groups D and E had no significant statistical difference from that of Group A (*P* = 1.0).

**Table 2:** Comparison of Objective indices of Image Quality Among Groups.

Item	Group A	Group B	Group C	Group D	Group E	F	P	P (A vs B)	P (A vs C)	P (A vs D)	P(A vs E)
CT _AOmean_	46.91±8.83(31.20–64.20)	49.21±9.08(32.50–78.51)	49.66±9.13(34.7–72.2)	47.06±8.80(31.0–62.20)	48.60±11.55(31.4–71.7)	0.46	0.76	1.00	1.00	1.00	1.00
Noise _AOmean_	14.93±3.14(10.10–23.00)	11.98±2.80(7.00–17.60)	12.22±2.22(7.20–17.40)	14.45±4.01(7.00–21.90)	15.09±3.64(9.4–23.70)	5.95	<0.001	0.01	0.03	1.00	1.00
SNR	3.27±0.90(1.70–5.27)	4.31±1.19(2.31–6.41)	4.19±1.11(2.59–7.02)	3.52±1.19(1.90–6.29)	3.42±1.23(1.32–6.96)	4.76	<0.001	0.01	0.03	1.00	1.00

### 2. Subjective Image Quality

The scores of subjective image quality showed a good inter-obsever consistency, and the Kappa values were as follows: central airway, 0.89; lung tissue, 0.88; mediastinum, 0.87; artefacts, 0.85; general image quality, 0.82.

**Fig. 1:**
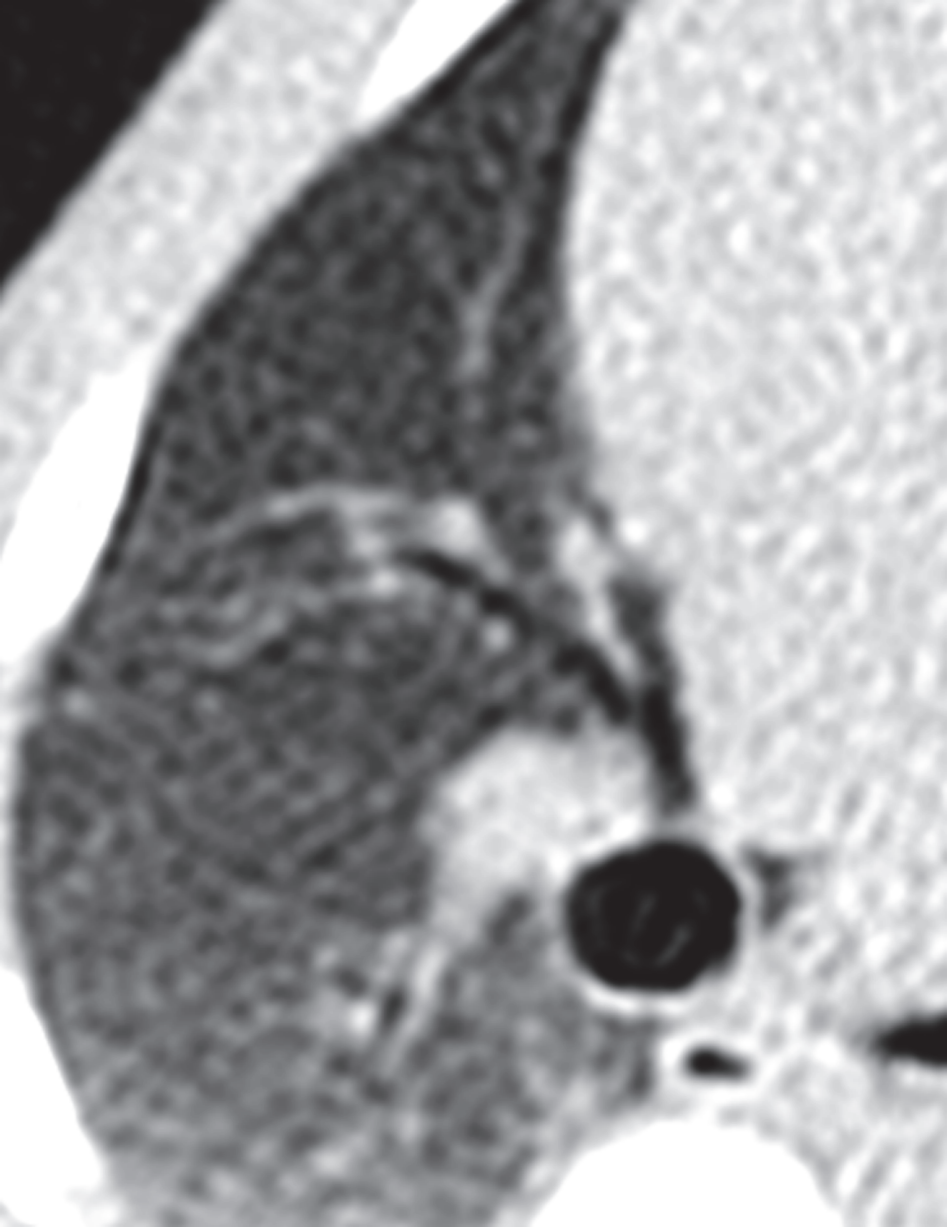
An infant pig weighing 8 kg. Fig. 1 depicts the image of lung window in Group A. Figs. 2–5 show the images in Group B-E. The image quality scores for Figs. 2 and 3 were both 5 points; furthermore, they were superior to that of Fig. 1, which scored 4 points. The image quality score for Fig. 4 was 4 points, which was identical to that of Fig. 1. However, Fig. 5 had poor image quality with extensive noise in the lung field. Small bronchi appeared unclear, so it was scored 3 points.

**Fig. 2:**
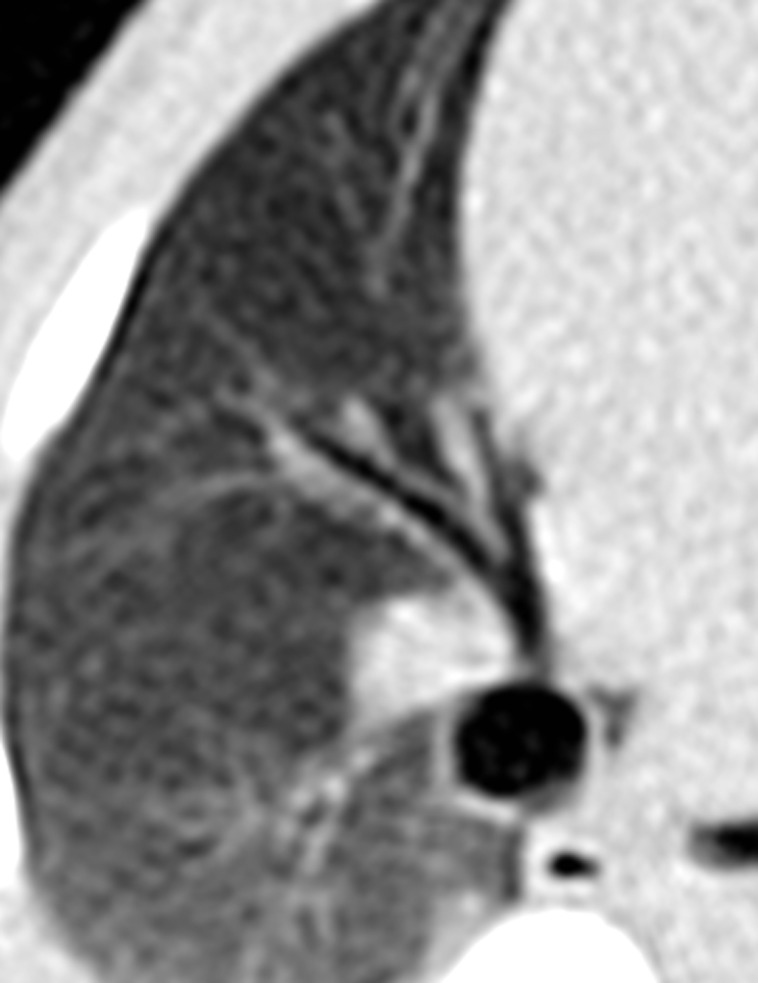
An infant pig weighing 8 kg. Fig. 1 depicts the image of lung window in Group A. Figs. 2–5 show the images in Group B-E. The image quality scores for Figs. 2 and 3 were both 5 points; furthermore, they were superior to that of Fig. 1, which scored 4 points. The image quality score for Fig. 4 was 4 points, which was identical to that of Fig. 1. However, Fig. 5 had poor image quality with extensive noise in the lung field. Small bronchi appeared unclear, so it was scored 3 points.

**Fig. 3:**
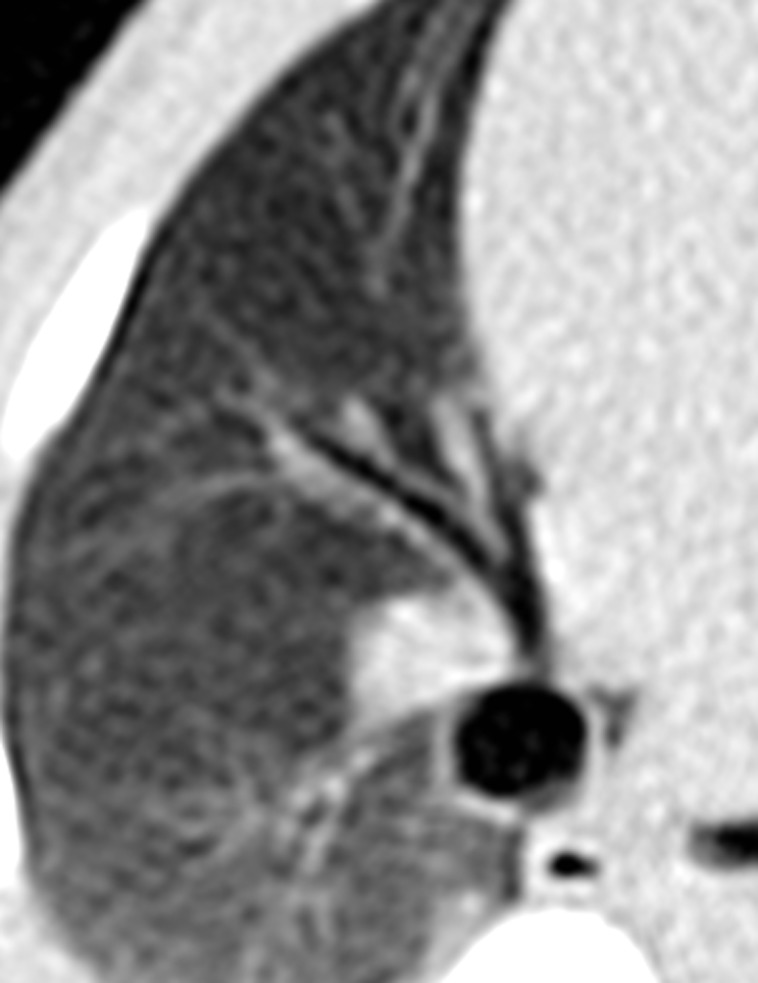
An infant pig weighing 8 kg. Fig. 1 depicts the image of lung window in Group A. Figs. 2–5 show the images in Group B-E. The image quality scores for Figs. 2 and 3 were both 5 points; furthermore, they were superior to that of Fig. 1, which scored 4 points. The image quality score for Fig. 4 was 4 points, which was identical to that of Fig. 1. However, Fig. 5 had poor image quality with extensive noise in the lung field. Small bronchi appeared unclear, so it was scored 3 points.

**Fig. 4:**
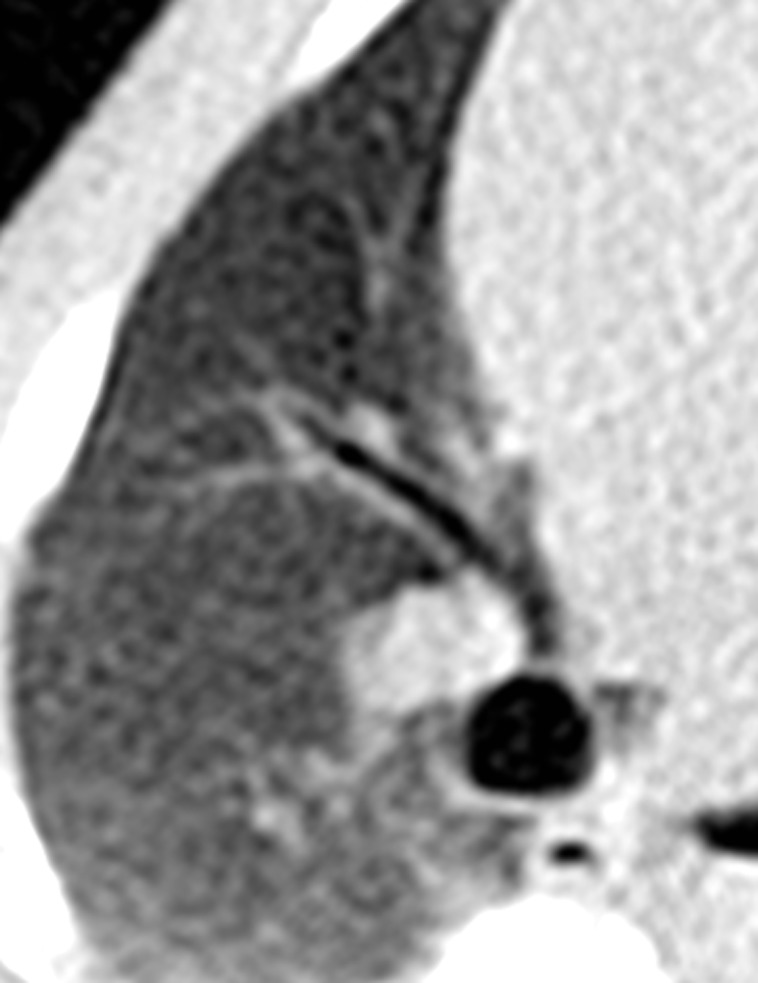
An infant pig weighing 8 kg. Fig. 1 depicts the image of lung window in Group A. Figs. 2–5 show the images in Group B-E. The image quality scores for Figs. 2 and 3 were both 5 points; furthermore, they were superior to that of Fig. 1, which scored 4 points. The image quality score for Fig. 4 was 4 points, which was identical to that of Fig. 1. However, Fig. 5 had poor image quality with extensive noise in the lung field. Small bronchi appeared unclear, so it was scored 3 points.

**Fig. 5:**
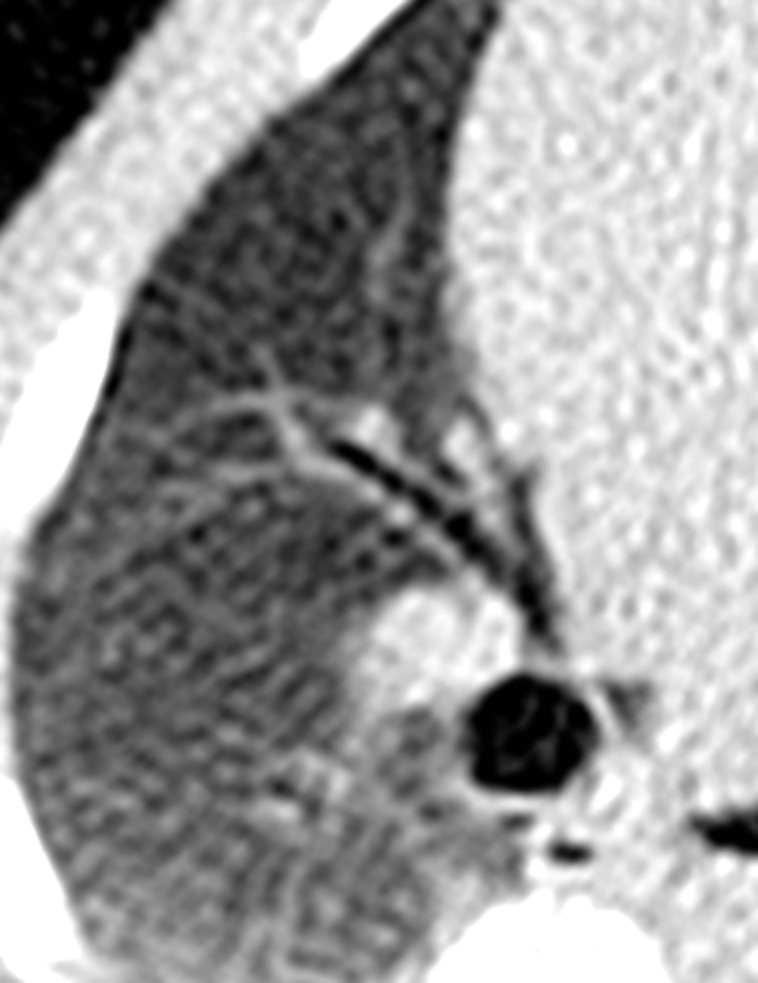
An infant pig weighing 8 kg. Fig. 1 depicts the image of lung window in Group A. Figs. 2–5 show the images in Group B-E. The image quality scores for Figs. 2 and 3 were both 5 points; furthermore, they were superior to that of Fig. 1, which scored 4 points. The image quality score for Fig. 4 was 4 points, which was identical to that of Fig. 1. However, Fig. 5 had poor image quality with extensive noise in the lung field. Small bronchi appeared unclear, so it was scored 3 points.

**Fig. 6:**
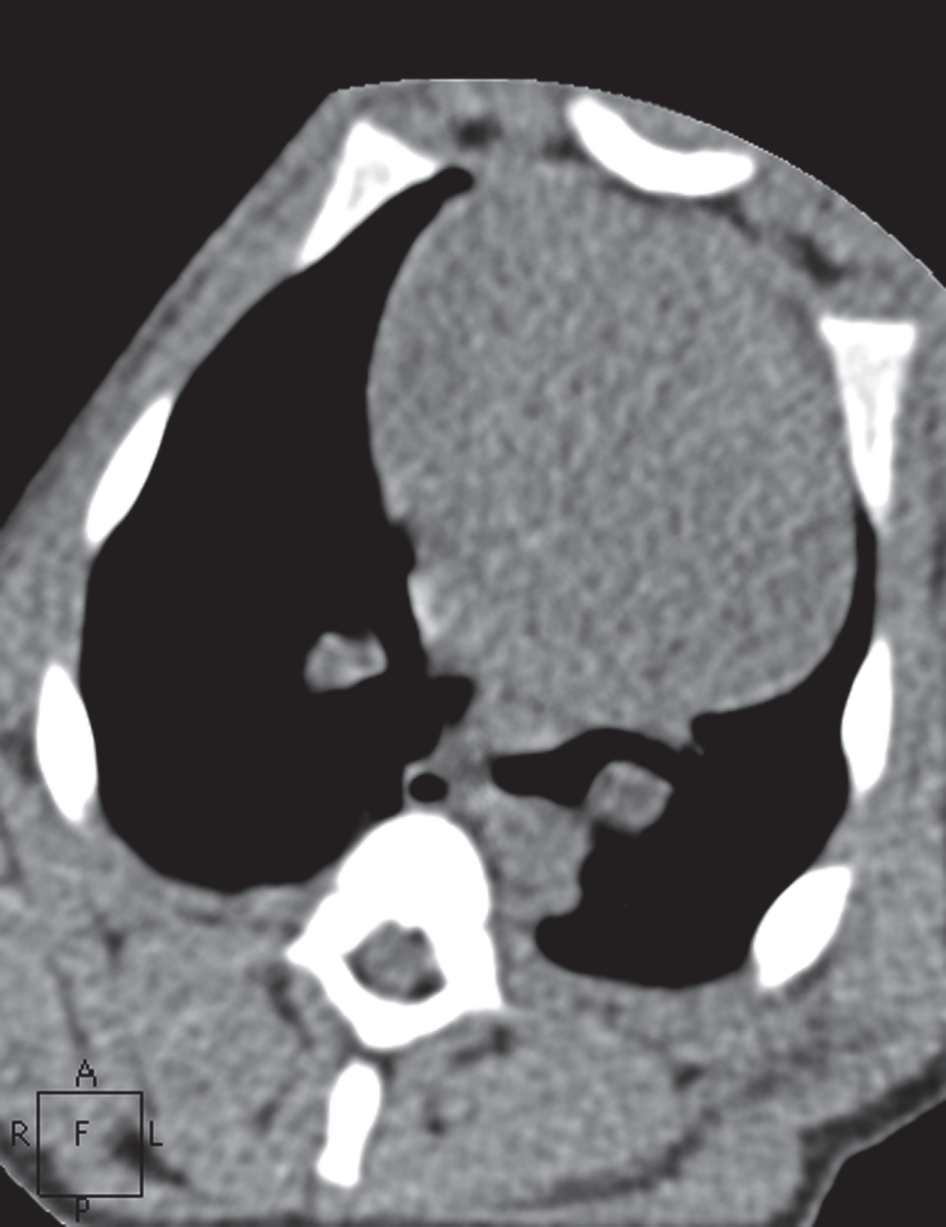
The images in mediastinal window of the same pig as Figs. 1–5. Figs. 6–10 show the images in Group A-E. The image quality scores for Figs. 7 and 8 were both 5 points. The image quality score for Fig. 9 was 4 points, which is comparable to Fig. 6. Fig. 10 had extensive noise, which was scored 2 points.

**Fig. 7:**
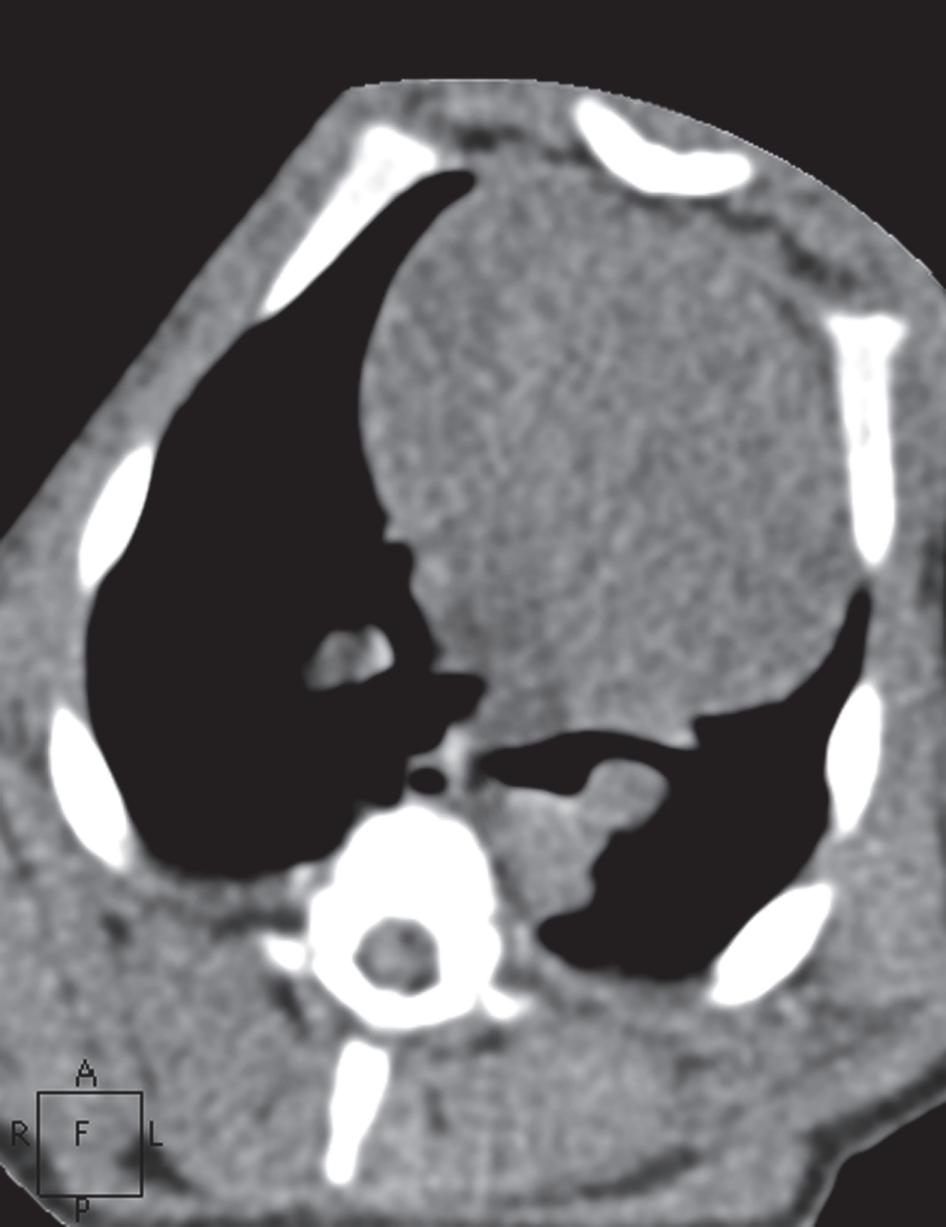
The images in mediastinal window of the same pig as Figs. 1–5. Figs. 6–10 show the images in Group A-E. The image quality scores for Figs. 7 and 8 were both 5 points. The image quality score for Fig. 9 was 4 points, which is comparable to Fig. 6. Fig. 10 had extensive noise, which was scored 2 points.

**Fig. 8:**
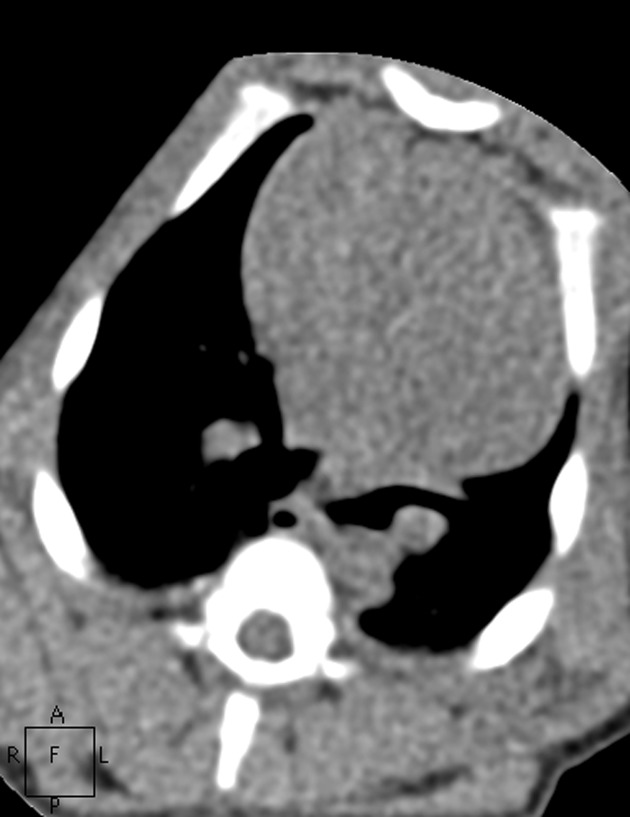
The images in mediastinal window of the same pig as Figs. 1–5. Figs. 6–10 show the images in Group A-E. The image quality scores for Figs. 7 and 8 were both 5 points. The image quality score for Fig. 9 was 4 points, which is comparable to Fig. 6. Fig. 10 had extensive noise, which was scored 2 points.

**Fig. 9:**
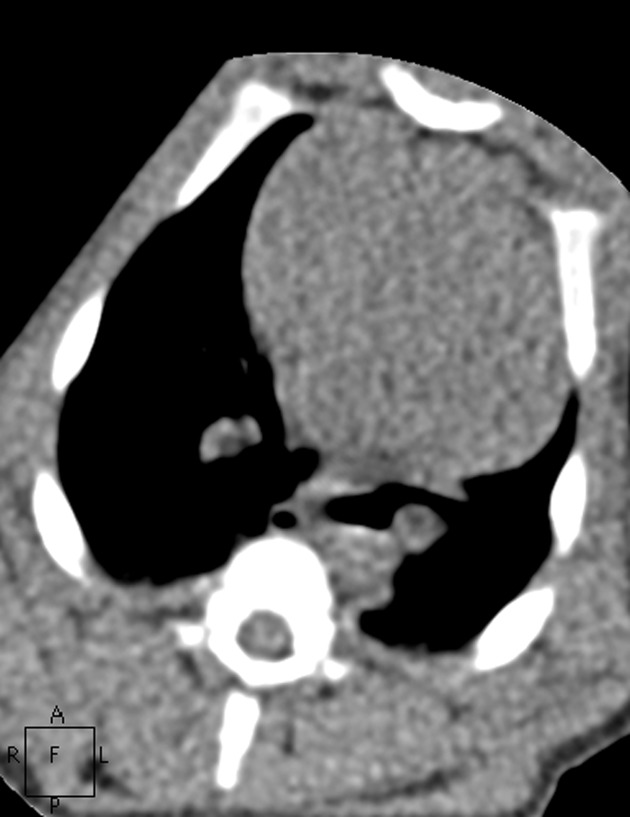
The images in mediastinal window of the same pig as Figs. 1–5. Figs. 6–10 show the images in Group A-E. The image quality scores for Figs. 7 and 8 were both 5 points. The image quality score for Fig. 9 was 4 points, which is comparable to Fig. 6. Fig. 10 had extensive noise, which was scored 2 points.

**Fig. 10:**
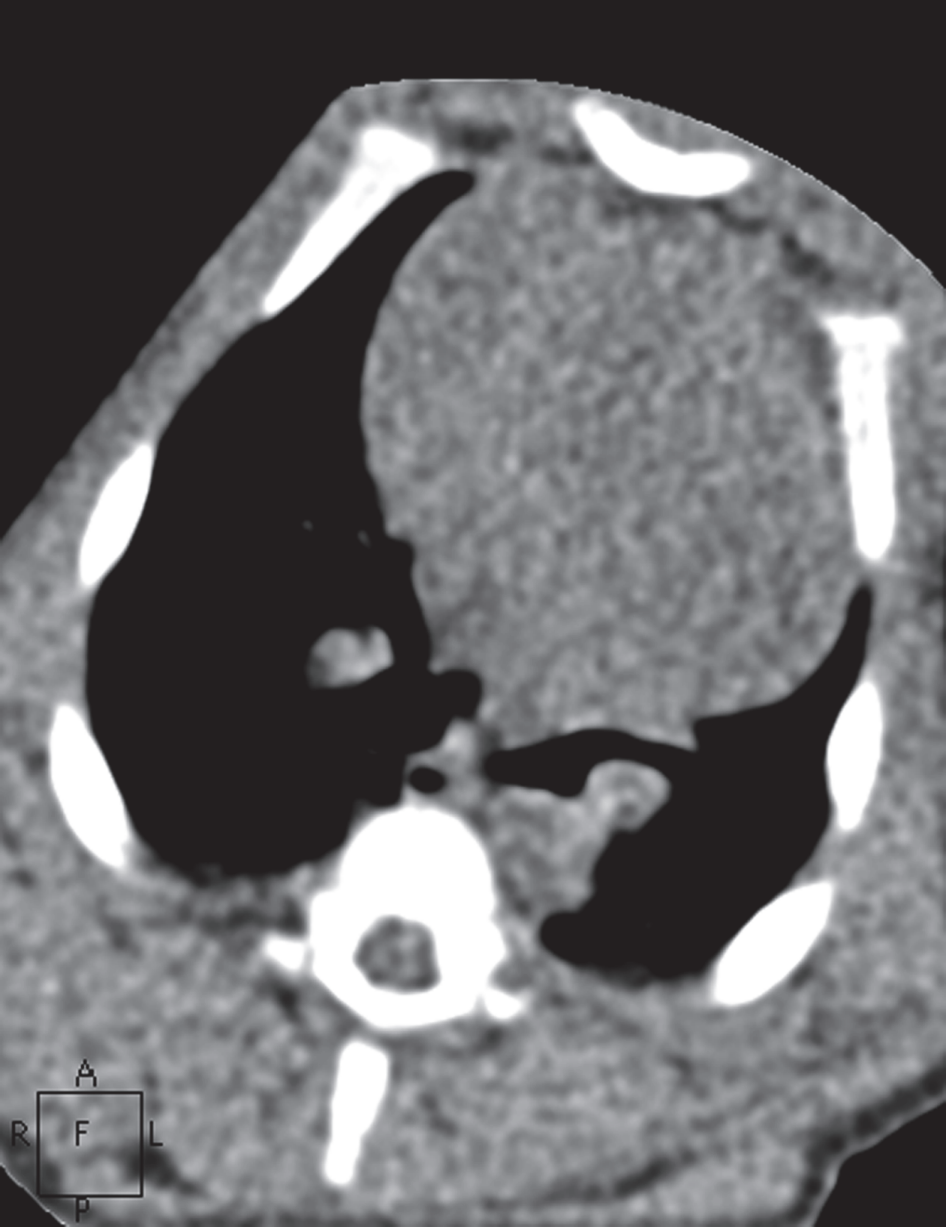
The images in mediastinal window of the same pig as Figs. 1–5. Figs. 6–10 show the images in Group A-E. The image quality scores for Figs. 7 and 8 were both 5 points. The image quality score for Fig. 9 was 4 points, which is comparable to Fig. 6. Fig. 10 had extensive noise, which was scored 2 points.

The scores of subjective image quality of all groups are presented in Table 3. The subjective scores of Groups B and C were higher than those of Group A. One exception was the score for lung tissue in Group C, which had no significant difference from that in Group A. Most of scores of subjective image quality of Group D were comparable to those of Group A (*P* > 0.05), except for general image quality scores, which were better in Group D. (Figs. 1–10). With further dose reduction, the scores of subjective image quality slightly decreased. In Group E, 5 pigs were scored 2 points due to the reduction of sharpness of the edge of the mediastinal structures. As a direct result, the diagnostic acceptability of general image quality (≥ 4 points) was reduced to 79.3% (23/27) for the group.

**Table 3:** Comparison of Subjective Image Quality Scores Among Groups.

Item	Group A	Group B	Group C	Group D	Group E	chi-square	P	P (A vs. B)	P (A vs. C)	P (A vs. D)	P (A vs. E)
Mediastinum	(0/0/13/14/0)	(0/0/0/5/22)	(0/0/0/25/2)	(0/0/13/14/0)	(0/5/22/0/0)	91.07	<0.001	<0.001	<0.001	1.00	<0.001
Central Airway	(0/0/0/24/3)	(0/0/0/0/27)	(0/0/0/16/11)	(0/0/0/26/1)	(0/0/8/19/0)	77.47	<0.001	<0.001	<0.013	0.16	<0.013
Lung Tissue	(0/0/0/25/2)	(0/0/0/0/27)	(0/0/0/17/10)	(0/0/0/26/1)	(0/0/8/19/0)	76.69	<0.001	<0.001	0.02	0.32	<0.013
Artifact	(0/0/0/26/1)	(0/0/0/0/27)	(0/0/0/16/11)	(0/0/0/26/1)	(0/0/8/19/0)	81.15	<0.001	<0.001	<0.01	1.00	<0.013
Image Quality	(0/0/4/23/0)	(0/0/0/0/27)	(0/0/0/16/11)	(0/0/0/26/1)	(0/0/4/9/14)	81.07	<0.001	<0.001	<0.001	0.03	<0.001

Note: (1/2/3/4/5) represented the scores of indexes for subjective evaluation.

Comparison of objective and subjective image quality scores among subgroups of Group D was shown in Table 4. There were no obvious statistical differences in the objective and subjective indices value among the subgroups of Group D.

**Table 4:** Comparison of Objective and Subjective Image Quality Scores Among subgroups of Group D.

Item	Small (n = 9)	Medium (n = 8)	Large (n = 10)	F/ chi-square	P
CT _AO mean_	48.27±10.57(31.30–62.20)_	48.29±8.85(31.00–57.50)	45.14±7.34(32.50–54.40)	0.38	0.69
Noise _AO mean_	13.02±3.24(7.00–17.60)	16.01±3.82(10.20–21.90)	14.41±3.89(7.23–18.20)	1.40	0.27
SNR	3.84±0.87(2.43–4.91)	3.21±1.22(1.90–5.64)	3.41±1.27(1.90–6.29)	0.71	0.50
Mediastinum	(0/0/3/6/0)	(0/0/4/4/0)	(0/0/6/4/0)	1.31	0.52
Central Airway	(0/0/0/9/0)	(0/0/0/7/1)	(0/0/0/10/0)	2.38	0.31
Lung Tissue	(0/0/0/9/0)	(0/0/0/7/1)	(0/0/0/10/0)	2.38	0.31
Artifact	(0/0/0/9/0)	(0/0/0/7/1)	(0/0/0/10/0)	2.38	0.31
Image Quality	(0/0/0/9/0)	(0/0/0/7/1)	(0/0/0/10/0)	2.38	0.31

Note: Small subgroup: Body Weight ≤ 5kg; Medium subgroup: 5 kg < Body Weight < 10kg; Large subgroup: 10kg ≤ BodyWeight ≤15kg

### 3. Radiation Dose

Table 5 presents the values of CTDI_vol_, DLP and ED in each group. Compared to Group A, the effective doses (ED) were reduced by: 23.2% in Group B; 48.2% in Group C;58.9% in Goup D; 64.3% in Group E. Group D had a subjective image quality comparable to that of Group A in which the ED was 0.20 ± 0.05 mSv. Table 6 presents the comparison of CTDI_vol_, DLP and ED of the subgroups of Group D and Group A.

**Table 5:** Comparison of Different Radiation Doses Among Groups.

Item	Group A	Group B	Group C	Group D	Group E	F	P
CTDI_vol_ (mGy)	1.12±0.26(0.60–1.60)	0.86±0.23(0.40–1.20)	0.58±0.18(0.10–1.00)	0.46±0.15(0.20–0.80)	0.40±0.17(0.20–1.00)	168.20	P<0.001
DLP(mGy.cm)	14.98±4.71(5.80–22.50)	11.84±3.92(4.10–17.00)	7.97±2.81(2.90–12.20)	6.19±2.36(2.30–10.50)	5.40±1.89(1.7–8.3)	113.66	P<0.001
ED(mSv)	0.48±0.10(0.23–0.63)	0.37±0.08(0.16–0.48)	0.25±0.06(0.11–0.34)	0.20±0.05(0.09–0.29)	0.17±0.04(0.07–0.23)	184.25	P<0.001

**Table 6:** Comparation of effective dose for three subgroups of Group D and Group A.

Subgroup	CTDI_vol_ (mGy)		DLP (mGy.cm)		ED (mSv)	
	Group A	Group D	Group A	Group D	Group A	Group D
Small (n = 9)	0.93±0.19(0.60–1.10)	0.35±0.09(0.20–0.50)	11.09±2.43(5.80–13.70)	4.28±1.00(2.30–5.60)	0.43±0.09(0.23–0.53)	0.17±0.44(0.09–0.22)
Medium (n = 8)	1.06±0.22(0.70–1.30)	0.43±0.11(0.30–0.60)	14.29±3.76(8.70–18.00)	5.87±1.65(3.50–8.00)	0.45±0.07(0.34–0.53)	0.19±0.03(0.14–0.22)
Large (n = 10)	1.38±0.15(1.10–1.60)	0.60±0.11(0.40–0.80)	20.13±1.98(16.00–22.50)	8.78±1.48(6.00–10.50)	0.56±0.06(0.45–0.63)	0.25±0.04(0.17–0.29)

Note: Small subgroup: Body Weight ≤ 5kg; Medium subgroup: 5 kg < Body Weight < 10kg; Large subgroup: 10kg ≤ BodyWeight ≤15kg

## Discussion

This is the first paper that evaluates of AIDR-3D in an infant pig model using 640-slice volume chest CT. Our study demonstrated that AIDR-3D can significantly reduce image noise and improve the image quality. The optimal scanning protocol was to combine AIDR-3D with ^SURE^Exposure 3D (SD17.5). This protocol resulted in a 58.9% reduction of radiation, compared to the routine dose.

AIDR-3D is a model-based iterative reconstruction algorithm recently approved and designed to work both in the raw data and image reconstruction domains. The AIDR-3D uses a scanner model and a statistical noise model in the raw data domain. The former is used to analyse the physical properties of the CT system at the time of the image acquisition, and the latter is used to characterize both the electronic and quantum noise patterns in the projection data domain. A sophisticated iterative technique operates in a continuous fashion to optimize reconstructions for the particular body region being scanned. This is accomplished by detecting and preserving sharp details while simultaneously smoothing the image. A terminating criterion based on the signal-to-noise ratio is determined to halt the iterative process. Finally, to maintain the noise granularity, a weighted blending was applied to the original reconstruction and the output of the iterative process. As a result, AIDR-3D reconstruction can increase the signal-to-noise ratio and improve the image quality; in addition, it can make the images appear more natural. The reconstruction speed for AIDR 3D processing is 32 frames per second, which is comparable to FBP reconstruction speed and can be met the requirements for clinical routine practice.

In contrast to other implementations of IR, AIDR-3D in conjunction with AEC system (^Sure^Exposure^3D^) [21] can automatically calculate the minimal amount of exposure that meets the need for diagnosis before scanning. By combining these two techniques, different noise indices (NI) can be selected as the reference values for target image quality, based on the particular examination requirements. It can be combined with other factors affecting the image quality, such as a convolution filter, thickness, artefact suppression method, and iterative algorithm in a comprehensive manner. It facilitates an accurate calculation of the scanning conditions necessary for the patient; thus, the optimal dose and scanning parameter combination for ensuring a good image quality can be obtained.

In this study, the analysis of objective indices showed that the CT values in low dose groups had no significant difference from those in group A. thus, it is in agreement with previous studies.[22–24] This finding indicates that iterative reconstruction can maintain the difference in absorbing X-rays of different tissues; thus, it can correctly distinguish the characteristics of tissue. Noise and SNR are the key indicators used to determine the image quality and anatomical details of the lungs and mediastinum. In comparison with Group A, the lower dose IR groups (groups B, C, D, and E) compensated for objective image noise caused by the dose reduction; furthermore, it maintained a higher SNR. This finding was in agreement with the results of the study by Miéville et al.[16] Their study showed that a proper dose reduction with iterative reconstruction could significantly reduce image noise and improve the SNR; furthermore, the image by iterative reconstruction at a low dose had a better image quality for visualizing small structures (e.g., vessels beneath the pleura and pulmonary lacerations).

The subjective image quality scores showed that Group D could achieve a comparable image quality to that in Group A (P > 0.05). For all subgroups in Group D, there was no significant difference in the subjective image quality scores. The adoption of ^Sure^Exposure3D technique means that it can automatically and correctly tune the mA value according to the pre-set noise index and the 3D body size in the pig model (e.g. diameters in antero-posterior and transverse directions). As a result, this technique can achieve the individualized dose reduction scheme while ensuring a similar level of image quality among the subgroups though there was a difference in subgroups for body weight and size. Thus, in conclusion, the scanning protocol of Group D can be taken as the preferred low dose scheme for a paediatric chest CT examination.

In Group E, 5 pigs were scored 2 points due to a lot of noise in mediastinum window, which shows that when the dose is reduced to 64.3% of the standard dose (Group A), the diagnostic information of partial mediastinal soft tissue can be missing. However, the evaluation indices for lung window(artefact, central airway, and lung tissue) in Group E all scored ≥ 4 points; thus, it indicated that the scheme of Group E can still be effectively applied to the examination of intrapulmonary lesions and to the follow-up CT of patients with mediastinal lesions.

From the image quality analysis, the optimal scanning condition for maintaining a diagnosable image quality was determined to be SD17.5 (2 mm for the thickness of layer, cross section) combined with AIDR-3D reconstruction in a strong noise reduction level. The CTDI_vol_ in Group D (SD17.5) was 0.46 ± 0.15 mGy (0.20–0.80 mGy); this signified a 58.9% reduction, compared with CTDI_vol_ of 1.12 ± 0.26 mGy (0.60–1.60 mGy) in Group A. In addition, this result was comparable to those of the applications of AIDR-3D and other IR techniques in the adult chest. [12,14] To our knowledge, the radiation dose (CTDI_vol_: 0.20–0.80mGy) corresponding to Group D was lower than that of previous similar studies (with comparable body weight).[4–10,25] Moreover, it was significantly lower than the radiation dose for the paediatric heart and chest suggested by two European standards in 2008 and 2010.[25,26] The 16 cm detector width of 640-slice volume CT can cover the entire paediatric chest with one axial rotation. The rotation speed of 0.5 s can instantly complete scanning without the need for breath-holding, which avoids re-examination typically seen in spiral scanning due to breath artefacts or over movements during acquisition. More importantly, it can prevent from extra radiation dose due to the re-examination.

This study has the following limitations. First, it was conducted on an animal model with a small sample size in each group; thus, the conclusions still require further validation with paediatric chest CT examinations. Second, since the young pigs have a similar cardiothoracic structure as that of human infants, pigs were selected on the basis of weight in order to imitate the thoracic conditions of human infants aged 0–3 years. However, we did not consider possible effects of the difference between the thoracic architecture (antero-posterior and transverse diameters of the thorax) of pigs and that of the chests of infants on the radiation dose. Thus, the dose results may be slightly different from the practical dose for the paediatric chest. Third, the natural maturity of young pigs differs from that of human infants; therefore, this difference might have some influence on the image quality evaluation. Finally, only normal infant pig models were used in this study; therefore, it is not possible to evaluate the manifestations of lesions by iterative reconstruction.

In conclusion, our study showed that AIDR-3D can significantly improve the image quality of chest CT in infant pigs. In the low dose group using AIDR-3D combined with ^SURE^Exposure 3D technique and setting the noise index to SD 17.5 can maintain the image quality comparable to the routine dose using a FBP reconstruction. It also shows that in this group AIDR 3D reconstruction has the ability to significantly reduce the radiation dose by 58.9%.
